# Potential beneficial effects of ixmyelocel-T in the treatment of atherosclerotic diseases

**DOI:** 10.1186/scrt346

**Published:** 2013-11-01

**Authors:** Kelly J Ledford, Nikki Murphy, Frank Zeigler, Ronnda L Bartel

**Affiliations:** 1 Aastrom Biosciences, Domino’s Farms, Lobby K, 24 Frank Lloyd Wright Drive, Ann Arbor, MI 48105, USA

## Abstract

**Introduction:**

Advanced atherosclerotic lesions are characterized by lipid accumulation, inflammation, and defective efferocytosis. An ideal therapy should address all aspects of this multifactorial disease. Ixmyelocel-T therapy, an expanded autologous multicellular therapy showing clinical promise in the treatment of diseases associated with advanced atherosclerosis, includes a novel population of M2-like macrophages. Here, we examine the macrophages of ixmyelocel-T and determine their ability to influx modified cholesterol in an atheroprotective manner, maintaining cholesterol homeostasis and preventing cellular dysfunction and death, ultimately promoting reverse cholesterol efflux.

**Methods:**

Approximately 50 ml of whole bone marrow was obtained from healthy donors and shipped overnight. Bone marrow mononuclear cells (BMMNCs) were produced by using density gradient separation and cultured for approximately 12 days to generate ixmyelocel-T. CD14+ cells were isolated from ixmyelocel-T via positive selection for analysis. Ixmyelocel-T and human leukemia monocyte (THP-1) cells were loaded with acetylated low-density lipoprotein (Ac-LDL) for analysis. Flow cytometry and immunofluorescence were used to examine Ac-LDL uptake, expression of cytokines was analyzed by enzyme-linked immunofluorescence assay (ELISA), and quantitative real-time PCR was used to analyze expression of cholesterol-transport genes. Both the *in vitro* cholesterol efflux assay and *in vivo* reverse cholesterol transport assay were used to examine cholesterol transport.

**Results:**

Ixmyelocel-T macrophages take up acetylated low-density lipoprotein and express the scavenger receptors *CD36* and *scavenger receptor-B1 (SR-B1)*. Ixmyelocel-T did not become apoptotic or proinflammatory after lipid loading. The cholesterol transporter genes *ABAC1* and *ABCG1* were both statistically significantly upregulated when ixmyelocel-T macrophages were loaded with cholesterol. Ixmyelocel-T also exhibited enhanced apolipoprotein A-I (ApoAI)-mediated cholesterol efflux. In addition, *in vivo* reverse cholesterol-transport assay demonstrated that ixmyelocel-T was able to efflux cholesterol in this model.

**Conclusions:**

Ixmyelocel-T macrophages influx modified cholesterol, remained anti-inflammatory in the face of lipid loading and inflammatory challenge, and displayed enhanced cholesterol efflux capabilities. These combined features suggest that this autologous multicellular therapy may exert beneficial effects in atherosclerotic diseases.

## Introduction

Atherosclerosis, a chronic inflammatory disease of the vessel wall, is a leading cause of death in developed countries [1]. Accumulation of lipid-loaded macrophage foam cells is a central feature in the formation of atherosclerosis. Macrophages, the most abundant cell type in atherosclerotic lesions, accumulate lipids, secrete inflammatory cytokines, and participate in all phases of atherosclerosis [2]. Maintaining macrophage cholesterol homeostasis is essential in preventing the pathogenesis of atherosclerosis [3]. Macrophage-scavenger receptors, such as scavenger receptor (SR)-A and CD36, mediate the unregulated uptake of modified LDL lipoproteins, which leads to foam cell formation [3-5]. Cholesterol efflux, which removes excess cholesterol from macrophages, is believed to be the major process involved in the regression of atherosclerotic lesions [6,7]. The net flux, or influx and efflux, of modified cholesterol plays a key role in maintaining cholesterol homeostasis in macrophages [8]. An imbalance between cholesterol uptake by scavenger receptors and efflux in macrophages is widely recognized as the underlying mechanism leading to progression of atherosclerosis [3,9].

Macrophage cholesterol efflux is considered to be protective against the development of atherosclerosis [10]. Free or unesterified cholesterol (FC) is toxic to macrophages and can lead to apoptosis [11]. To prevent cell death, cholesterol is stored as cholesterol esters (CEs) within macrophage foam cells and effluxed as FC. Cholesterol efflux is mediated by the ATP-binding cassette transporters ABCA1 and ABCG1 [3]. ABCA1 regulates cholesterol efflux to lipid-free apolipoprotein A-I (apoA-I), and ABCG1 regulates cholesterol efflux to high-density lipoprotein (HDL) [12,13]. Both ABCA1 and ABCG1 prevent cholesterol accumulation and play an important role in maintaining macrophage cholesterol homeostasis. Excess ingested cholesterol is esterified by acyl-CoA:cholesterol acyl-transferase 1 (ACAT-1) and stored as lipid droplets in macrophages [3]. Cholesterol ester hydrolase (CEH) releases free cholesterol from stored CE, allowing it to be removed from macrophages through cholesterol efflux [5]. Studies have shown that macrophages with high CEH activity accumulate fewer CEs. Promoting macrophage cholesterol efflux could prevent progression or induce regression of atherosclerosis [11].

Macrophages are a diverse population of cells that adapt and respond to a variety of signals, including cytokines and microbial products [14]. Classically activated macrophages, or M1, produce proinflammatory cytokines and kill microorganisms [14], whereas alternatively activated macrophages, or M2, regulate the inflammatory response by producing antiinflammatory cytokines, scavenging debris, and promoting tissue repair [14-18]. Both populations are considered extremes of a wide variety of macrophage phenotypes [19]. Atherosclerotic plaques have been found to contain a mixture of M1 and M2 macrophages [20,21]. Recent studies have suggested that M1 macrophages are dominant in both initial and developing atherosclerotic lesions, whereas M2 macrophages are associated with lesion regression [20,22,23]. It is thought that M2 macrophages may help prevent lesion progression by secreting antiinflammatory cytokines, such as interleukin (IL)-10, and efficiently removing apoptotic cells [20].

Ixmyelocel-T is an expanded autologous multicellular therapy containing a combination of cell types derived from cultured bone marrow mononuclear cells [24]. Ledford KJ, Murphy N, Zeigler F, Bartel RL (unpublished data) found that ixmyelocel-T contains a population of unique M2-like macrophages that are characterized by secretion of the antiinflammatory cytokines IL-10 and IL-1ra, with minimal secretion of the proinflammatory cytokines tumor necrosis factor-alpha (TNF-α) and IL-12 after inflammatory challenge (Ledford KJ, Murphy N, Zeigler F, Bartel RL, unpublished data). Furthermore, these macrophages have demonstrated efferocytosis (that is, efficient removal of apoptotic cells) (Ledford KJ, *et al*., unpublished data). Both of these characteristics have been hypothesized to have beneficial effects in the treatment of atherosclerosis: IL-10 inhibits the secretion of inflammatory cytokines from other cells [20], and efferocytosis is considered atheroprotective [20]. Defective efferocytosis has been shown to promote atherosclerosis [25].

The goal of this investigation was to examine further ixmyelocel-T macrophages and determine the ability of these cells to influx/efflux modified cholesterol in an atheroprotective manner, by maintaining cholesterol homeostasis and preventing cellular dysfunction and death, ultimately promoting reverse cholesterol efflux.

In this study, THP-1 macrophages were used as a control in these investigations. THP-1 cells are commonly used as a model to delineate macrophage gene expression and functions associated with modified cholesterol loading and have been established as an effective model to study the effects of cholesterol homeostasis [26-29].

## Methods

### Cell culture

For the generation of ixmyelocel-T, commercially available bone marrow aspirates (Lonza, Walkersville, MD, USA) were obtained from healthy donors under informed consent. A small amount (about 50 ml) of whole bone marrow was obtained through needle aspiration of the posterior iliac crest, and stored in heparinized tubes. The mononuclear cell fraction was obtained through an automated, closed-system, Ficoll-based density gradient centrifugation separation process. The isolated mononuclear cells were then transferred to a sterile, single-use cell cassette [30]. This proprietary system controlled temperature, culture-medium exchange, and gas exchange during the culture period. After approximately 12 days, the cells were washed and harvested from the cassette by a multistep, automated process. The cells were then used for experimental study.

THP-1 cells were obtained from ATCC (Manassas, VA, USA) and cultured in RPMI 1640 (ATCC) containing 10% fetal bovine serum and 2 m*M* L-glutamine, according to the manufacturer’s instructions. THP-1 monocytes were differentiated into macrophages by using 200 n*M* phorbol myristate acetate (InvivoGen, San Diego, CA, USA) for 3 days, after which they were used in the following experiments.

### CD14^+^ cell purification

CD14^+^ cells were isolated from ixmyelocel-T by positive selection by using MACS beads (Miltenyi Biotec, Bergisch Gladbach, Germany), according to the manufacturer’s directions. After positive selection, the CD14^+^ ixmyelocel-T macrophages were transferred to six-well culture plates for subsequent experiments.

### Dil-Ac-LDL immunostaining

For fluorescent imaging, CD14^+^ ixmyelocel-T and THP-1 macrophages were plated in glass-chamber slides and allowed to adhere. Cells were loaded with 10 μg/ml Dil-Ac-LDL in serum-free media containing 0.2% bovine serum albumin (BSA; fatty acid free; Sigma-Aldrich, St. Louis, MO, USA). After 24 hours, the cells were washed with phosphate-buffered saline (PBS) and fixed in 10% formalin for 10 minutes. Counterstaining was performed with DAPI to visualize nuclei. Fluorescent images were visualized by using a Nikon Eclipse 80i (Nikon, Melville, NY, USA) equipped with an EXi Aqua Bio-Imaging Microscopy Camera (Q Imaging, Surrey, BC, Canada). For flow-cytometry quantification of ac-LDL uptake, ixmyelocel-T and THP-1 macrophages were loaded with 10 μg/ml Dil-Ac-LDL in serum-free media. DiI-AcLDL uptake was analyzed with flow cytometry with at least 10,000 events acquired for each sample. DiI-AcLDL uptake was quantified by mean fluorescent intensity, which was calculated by dividing the value of positive gated events by the value of the negative population.

### Cytokine secretion

Enzyme-linked immunosorbent assay kits were used to determine the concentrations of IL-1β, TNF-α, IL-6, and monocyte chemoattractant protein (MCP)-1 (R&D Systems, Minneapolis, MN, USA). In brief, CD14^+^ ixmyelocel-T and THP-1 macrophages were loaded with 50 μg/ml acetylated low-density lipoprotein (Ac-LDL; Fisher Scientific, Pittsburg, PA, USA) for 24 hours in serum-free media containing 0.2% BSA (fatty acid free). After 24 hours, the cells were washed with PBS and treated with lipopolysaccharide (LPS; 0.1 μg/ml) overnight. Supernatants were then collected and assayed by following manufacturer’s directions. For cytokine analysis, the values were normalized to cell number.

### Apoptosis assay

Apoptosis was measured in both ixmyelocel-T and THP-1 macrophages loaded with Ac-LDL. In brief, cells were seeded in 96-well plates and loaded with 100 μg/ml Ac-LDL for 24 hours in serum-free media containing 0.2% BSA (fatty acid free). The concentration of Ac-LDL used to induce apoptosis was based on our preliminary dose-finding study, which demonstrated that loading with 100 μg/ml Ac-LDL induced apoptosis in THP-1 macrophages. Induction of apoptosis was examined by measuring the caspase-3 and caspase-7 activity with the Caspase-Glo 3/7 Assay (Promega). Luminescence units were measured by using a SpectraMax plate reader (Molecular Devices, Sunnyvale, CA, USA), and values were expressed as a percentage relative to the values obtained from the control group (THP-1 macrophages).

### Cholesterol-efflux assay

Ixmyelocel-T and THP-1 macrophages were plated at 0.5 × 10^6^ cells/well, labeled with ^3^H-cholesterol for 24 hours, and allowed to equilibrate overnight. Cholesterol efflux was measured by using 20 μg/ml apoA-I and 20 μg/ml HDL_3_ in acceptor media after 4 hours. The efflux media and cells were analyzed to determine the percentage of cholesterol release.

### Reverse cholesterol transport

Experiments were performed in severe combined immunodeficient (SCID) mice (*n* = 10). In brief, ^3^H-cholesterol-labeled ixmyelocel-T (*n* = 3) and J774 cells (3 to 5 × 10^6^) 0.5 ml MEM-HEPES were injected intraperitoneally. Blood was collected at 24 and 48 hours, and plasma was used for scintillation counting. Feces were collected continuously for 48 hours and used for scintillation counting.

### Real-time PCR

Both CD14^+^ ixmyelocel-T and THP-1 macrophages were loaded with 50 μg/ml Ac-LDL for 24 hours in serum-free media containing 0.2% BSA (fatty acid-free). After 24 hours, the cells were washed with PBS and harvested for RNA isolation. For real-time PCR, total RNA was extracted with an RNeasy Mini kit (Qiagen, Valencia, CA, USA), and 1 μg of RNA was reverse transcribed by using the high-capacity cDNA reverse transcription kit per the manufacturer’s directions (Applied Biosciences, Carlsbad, CA, USA). Relative levels of target-gene expression were measured on the 7500 Real-Time PCR system (Applied Biosystems). FAM-based Taqman Gene Expression Assay Mix (Applied Biosystems) specific for each gene of interest and Taqman Universal Master Mix (Applied Biosystems) were used. Relative quantification PCR analysis was performed by using the ABI 7500 Software (Applied Biosystems). The relative amount of cDNA was calculated by normalization to GAPDH.

### Statistical analysis

Paired *t* tests were performed to compare results. A *P* value less than 0.05 was considered statistically significant. Data are reported as mean ± SEM.

## Results

### Ixmyelocel-T macrophages influx modified cholesterol

**Figure 1:**
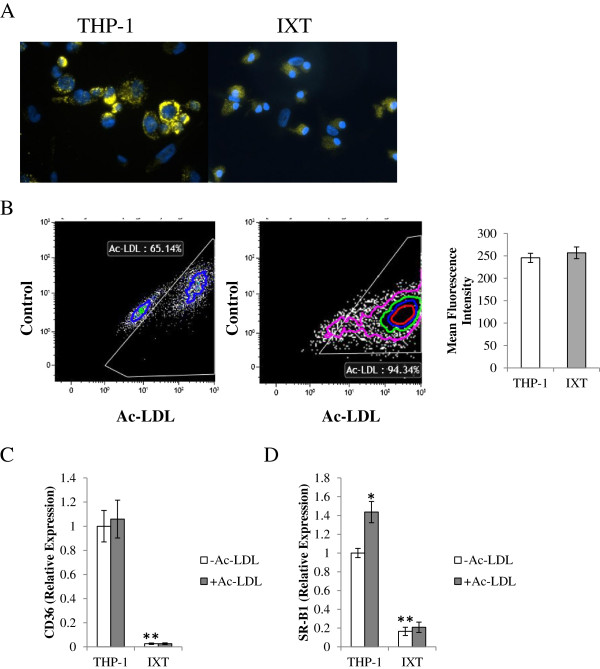
**Ixmyelocel-T macrophages influx modified cholesterol. (A)** Fluorescent microscopy images and **(B)** flow-cytometry analysis (*n* ≥ 5) of ixmyelocel-T and THP-1 macrophages loaded with Dil-Ac-LDL. Quantitative real-time PCR gene-expression analysis of scavenger receptors normalized to GAPDH (*n* ≥ 5). Expression of **(C)** CD36 and **(D)** SCARB1 in THP-1 and ixmyelocel-T macrophages before and after lipid loading. Values are presented as mean ± SEM relative to control; ^*^P < 0.01 versus THP-1 -Ac–LDL, ^**^P < 0.001 versus THP-1 -Ac–LDL. Magnification: 40×. Dil-Ac-LDL, low-density lipoprotein from human plasma, acetylated, Dil complex; PCR, polymerase chain reaction; GAPDH, glyceraldehyde 3-phosphate dehydrogenase; SCARB1, scavenger receptor class B type I; SEM, standard error of the mean; Ac-LDL, acetylated low-density lipoprotein.

The ability to ingest modified cholesterol and the degree of Ac-LDL accumulation was examined with fluorescent microscopy and flow cytometry by using Dil-Ac-LDL (Figure 1). Fluorescent microscopy revealed that the THP-1 macrophages loaded with Dil-Ac-LDL appeared to be loaded with large lipid droplets (Figure 1A). Ixmyelocel-T macrophages loading with Dil-Ac-LDL appeared to be loaded with smaller lipid droplets (Figure 1A). Flow cytometry revealed that both ixmyelocel-T and THP-1 ingest similar amounts of Dil-Ac-LDL (257 ± 13 versus 246 ± 10 mean fluorescent intensity; *P* = 0.26; compared with THP-1; Figure 1B). These results provide evidence that ixmyelocel-T macrophages do influx modified cholesterol. These results also suggest that ixmyelocel-T macrophages do not store large droplets, or pools, of cholesterol.

To understand further the mechanisms of Ac-LDL uptake, real-time PCR was used to examine expression of the modified lipid-scavenger receptors *CD36* and *scavenger receptor (SR)-B1* before and after lipid loading with Ac-LDL. No statistically significant increase in *CD36* expression was found after lipid loading in either ixmyelocel-T or THP-1 macrophages. THP-1 cells exhibited increased expression of *SR-B1* after lipid loading (1.44 ± 0.11 versus 1 ± 0.05-fold change, *P* < 0.01, compared with THP-1-Ac–LDL; Figure 1D), whereas no change in expression of *SR-B1* occurred in ixmyelocel-T macrophages treated with Ac-LDL (0.16 ± 0.04 versus 0.21 ± 0.05-fold change; *P* = NS compared with ixmyelocel-T + Ac-LDL; Figure 1D). These data suggest that ixmyelocel-T macrophages influx Ac-LDL through scavenger receptors that ingest modified lipids.

### Ixmyelocel-T does not become apoptotic after lipid loading

**Figure 2:**
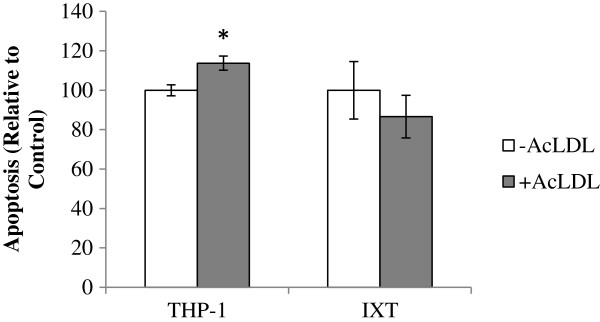
**Ixmyelocel-T is not sensitive to modified cholesterol-induced lipotoxicity; percentage of apoptosis after cholesterol loading.** Values are presented as mean ± SEM relative to control; ^*^P < 0.01 versus THP-1 -Ac–LDL. SEM, standard error of the mean; Ac-LDL, acetylated low-density lipoprotein.

To determine if lipid loading resulted in apoptosis, both ixmyelocel-T and THP-1 macrophages were loaded with 100 μg/ml Ac-LDL for 24 hours. Apoptosis was then analyzed by using a luminometric caspase 3/7 assay, which examines activation of caspases-3 and -7. As shown in Figure 2, THP-1 macrophages exhibited a statistically significant higher level of Ac-LDL-induced apoptosis (114% ± 3.58% versus 100% ± 3.58% of control; *P* < 0.01 compared with THP-1 -Ac–LDL; Figure 2), than ixmyelocel-T (87% ± 10.78% versus 100% ± 14.58% of control; *P* = NS compared with ixmyelocel-T -Ac–LDL; Figure 2).

### Ixmyelocel-T secretes minimal amounts of pro-inflammatory cytokines after lipid loading

When macrophages are unable to maintain cholesterol homeostasis because of ineffective cholesterol efflux, a proinflammatory response is generated [5]. Therefore, IL-1β, TNF-α, IL-6, and MCP-1 secretion was measured in lipid-loaded THP-1 and ixmyelocel-T macrophages before and after LPS stimulation (Table 1). When stimulated with LPS, THP-1 macrophages secreted significantly elevated amounts of IL-1β, TNFα, IL-6, and MCP-1 (Table 1). When ixmyelocel-T macrophages were stimulated with LPS, a statistically significant increase in IL-6 was found; however, the level of IL-6 is lower compared with those from THP-1 macrophages (Table 1). Overall, THP-1 macrophages secreted significantly higher amounts of the inflammatory cytokines after LPS stimulation compared with ixmyelocel-T macrophages. THP-1 macrophages exhibited a 514-fold increase for IL-1β, a 497-fold increase for TNF-α, a 162-fold increase for IL-6, and a 32-fold increase for MCP-1 (Table 1). However, ixmyelocel-T macrophages exhibited a fivefold increase for IL-1β, a twofold increase for TNF-α, a threefold increase for IL-6, and a 1.5-fold increase for MCP-1 (Table 1). These data demonstrate that ixmyelocel-T macrophages remain antiinflammatory even after lipid loading.

**Table 1:** Ixmyelocel-T secretes minimal amounts of proinflammatory cytokines after lipid loading

	**THP-1 (-LPS)**	**THP-1 (+LPS)**	**IXT (-LPS)**	**IXT (+LPS)**
**IL-1β**	25.7 ± 6	13,213 ± 269^a^	2.2 ± 0.8^b^	10 ± 1.9^a^
**TNF-α**	135 ± 30	67,063 ± 8,539^a^	86 ± 54	151 ± 80^a^
**IL-6**	97 ± 19	15,733 ± 1,874^a^	769 ± 353^c^	2,376 ± 551^ad^
**MCP-1**	940 ± 120	29,611 ± 2,713^a^	5,575 ± 1,912^c^	8,480 ± 2,491^a^

Cytokines were quantified in supernatants from lipid-loaded ixmyelocel-T and THP-1 macrophages treated with and without LPS (*n* ≥ 6). The amount of cytokine expressed was normalized to the cell number of each sample. Values are presented as mean ± SEM relative to control. ^a^*P* < 0.001 versus THP-1 - LPS; ^b^*P* < 0.01 versus THP-1 - LPS. ^c^*P* < 0.05, LPS, lipopolysaccharide; SEM, standard error of the mean; IXT, ixmyelocel-T. ^d^*P* < 0.05 versus IXT -LPS.

### Ixmyelocel-T displays enhanced cholesterol-efflux capacity

**Figure 3:**
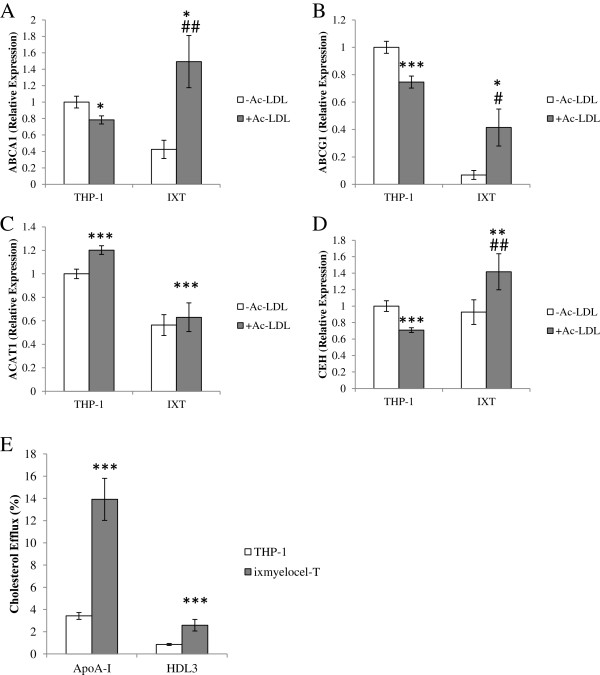
**Ixmyelocel-T displays enhanced cholesterol-efflux capacity.** Quantitative real-time PCR gene-expression analysis of scavenger receptors normalized to GAPDH (*n* ≥ 5). Expression of **(A)** ABCA1, **(B)** ABCG1, **(C)** ACAT1, and **(D)** CEH in THP-1 and ixmyelocel-T macrophages before and after lipid loading. **(E)** Cholesterol efflux of both THP-1 macrophages and ixmyelocel-T to the lipid acceptors ApoA-I and HDL3 (*n* ≥ 4). Values are presented as mean ± SEM relative to control; ^*^P < 0.05, ^**^P < 0.01, ^***^P < 0.001 versus THP-1 -Ac–LDL; ^#^*P* < 0.05, ^##^*P* < 0.01 versus IXT -Ac–LDL. PCR, polymerase chain reaction; GAPDH, glyceraldehyde 3-phosphate dehydrogenase; ABC, ATP-binding cassette; ACAT, acyl-CoA:cholesterol acyl-transferase; CEH, cholesterol ester hydrolase; ApoA-I, apolipoprotein A-I; HDL, high-density lipoprotein; SEM, standard error of the mean; Ac-LDL, acetylated low-density lipoprotein.

With real-time PCR, expression of key genes involved in cholesterol efflux (for example, *ABCA1* and *ABCG1*) was examined with and without cholesterol loading. There was a statistically significant increase in expression of *ABCA1* in ixmyelocel-T macrophages after cholesterol loading (1.49 ± 0.32 versus 0.42 ± 0.11-fold change; *P* < 0.01 compared with ixmyelocel-T -Ac–LDL; Figure 3A), whereas *ABCA1* was significantly decreased in THP-1 macrophages loaded with cholesterol (0.78 ± 0.05 versus 1 ± 0.07-fold change, *P* < 0.05 compared with THP-1 -Ac–LDL; Figure 3A). Additionally, ixmyelocel-T macrophages expressed significantly more *ABCA1* after lipid loading compared with THP-1 macrophages (1.49 ± 0.32 versus 0.78 ± 0.05-fold change, *P* < 0.05 compared with THP-1 + Ac-LDL; Figure 3A). Expression of *ABCG1* was significantly increased in ixmyelocel-T macrophages after cholesterol loading (0.41 ± 0.13 versus 0.07 ± 0.03-fold change, *P* < 0.05 compared with ixmyelocel-T -Ac–LDL; Figure 3B), whereas *ABCG1* was significantly decreased in THP-1 macrophages loaded with cholesterol (0.75 ± 0.04 versus 1 ± 0.04-fold change, *P* < 0.001 compared with THP-1 -Ac–LDL; Figure 3B).

*ACAT1* and *CEH* were also examined in both cell populations. Ixmyelocel-T macrophages expressed significantly lower *ACAT1* compared with THP-1 macrophages (0.56 ± 0.08 versus onefold ± 0.03-fold change, p < 0.001 compared with THP-1 -Ac–LDL; Figure 3C). After lipid loading, no change was seen in *ACAT1* expression in ixmyelocel-T macrophages (0.63 ± 0.11-fold versus 0.56- ± 0.08-fold change, *P* = NS compared with ixmyelocel-T -Ac–LDL; Figure 3C), whereas THP-1 macrophages expressed significantly elevated levels of *ACAT1* (1.2-fold ± 0.03-fold versus onefold ± 0.03-fold change, *P* < 0.001 compared with THP-1 -Ac–LDL; Figure 3C). After lipid loading, a statistically significant increase in *CEH* expression was noted in ixmyelocel-T macrophages (1.42 ± 0.22-fold versus 0.93 ± 0.15-fold change, *P* < 0.01, compared with ixmyelocel-T -Ac–LDL; Figure 3D), whereas THP-1 macrophages expressed significantly decreased levels of *CEH* (0.71 ± 0.03-fold versus 1-fold ± 0.06-fold change, *P* < 0.001 compared with THP-1 -Ac–LDL; Figure 3D). Because these genes, which are involved in cholesterol efflux, were upregulated in ixmyelocel-T, an *in vitro* cholesterol efflux assay was used to determine the ability of both cells to efflux cholesterol. Apolipoprotein A-I (ApoA-I)-mediated cholesterol efflux was significantly higher in ^3^H-cholesterol-AcLDL-loaded ixmyelocel-T compared with THP-1 macrophages (13.91% ± 1.90% versus 3.42% ± 0.31%, *P* < 0.001 compared with THP-1; Figure 3E). HDL_3_-mediated cholesterol efflux was significantly higher in ^3^H-cholesterol-AcLDL-loaded ixmyelocel-T compared with THP-1 macrophages (2.58% ± 0.052% versus 0.85% ± 0.09%, *P* < 0.01 compared with THP-1; Figure 3E). Collectively, these data indicate that ixmyelocel-T macrophages exhibit enhanced capacities to efflux cholesterol.

### Ixmyelocel-T promotes reverse cholesterol transport

**Figure 4:**
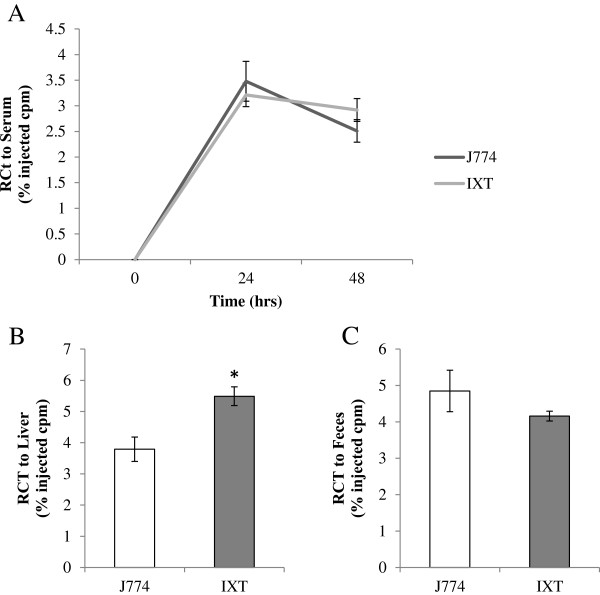
**Ixmyelocel-T promotes reverse cholesterol transport. ***In vivo* cholesterol efflux was examined in SCID mice after intraperitoneal injections of either ^3^H-cholesterol-loaded J774 cells or ixmyelocel-T. **(A)** Plasma ^3^H-cholesterol levels after 24 and 48 hours, **(B)**^3^H-tracer found in the liver, and **(C)**^3^H-tracer found in the feces after 48 hours (*n* ≥ 3 per group). Values are presented as mean ± SEM relative to control, ^*^P < 0.05 versus J774. SCID, severe combined immunodeficient; SEM, standard error of the mean.

The classic reverse cholesterol transport (RCT) pathway involves cholesterol efflux from macrophage foam cells to cholesterol acceptors and the excretion of cholesterol into the feces [11,31]. The *in vivo* RCT assay was used to determine whether ixmyelocel-T macrophages are capable of effluxing cholesterol *in vivo*. As shown in Figure 4, ixmyelocel-T is capable of promoting RCT *in vivo*. After injection of ^3^H-cholesterol-labeled ixmyelocel-T into the peritoneal cavity of SCID mice, plasma ^3^H-cholesterol levels were similar to the control cell line J774 cells after both 24 hours (3.21% ± 0.23% versus 3.48% ± 0.39% injected cpm, *p* = NS compared with J774; Figure 4A) and 48 hours (2.92% ± 0.22% versus 2.51% ± 0.22% injected cpm, *P* = NS compared with J774; Figure 4A). Statistically significantly more ^3^H-tracer was found in the livers of mice injected with ixmyelocel-T compared with the J774 cells (5.49% ± 0.30% versus 3.79% ± 0.39% injected cpm, *P* < 0.05 compared with J774; Figure 4B), and no difference was found in the feces (4.16% ± 0.14% versus 4.85% ± 0.57% injected cpm, *P* = NS compared with J774; Figure 4C). These data demonstrate that ixmyelocel-T is able to efflux cholesterol *in vivo*, promoting cholesterol efflux in this model.

## Discussion

The current study provides evidence that ixmyelocel-T macrophages influx and efflux modified cholesterol in an atheroprotective manner. The characteristics of ixmyelocel-T macrophages help maintain cellular cholesterol homeostasis, preventing cellular dysfunction and death, and ultimately promoting reverse cholesterol efflux. These data point to a potential new innovative application of ixmyelocel-T in the treatment of atherosclerotic diseases where massive amounts of modified cholesterol are present. Not only are ixmyelocel-T macrophages less susceptible to becoming foam cells, but they also appear to house the biologic machinery necessary to promote RCT, which has the potential to prevent progression or induce regression of atherosclerosis.

Uncontrolled accumulation of modified lipids in macrophages is a continuous process that contributes to all stages of atherosclerosis. Expression of scavenger receptors on macrophages is not downregulated by the accumulation of intracellular lipids, resulting in uncontrolled lipid accumulation and eventual irreversible cell damage and death [32]. This study and previously reported data (Ledford *et al*., unpublished data) demonstrated that ixmyelocel-T macrophages express relatively low levels of surface receptors involved in the uptake of modified cholesterol. Specifically, previous data demonstrated that ixmyelocel-T macrophages expressed lower levels of scavenger receptors involved in modified cholesterol uptake compared with M2 macrophages. In addition, ixmyelocel-T macrophages did not upregulate expression of these surface receptors in the presence of modified lipids. Fluorescence microscopy and flow cytometry demonstrated that ixmyelocel-T macrophages exhibited accumulation of modified LDL, showing that they do indeed influx modified cholesterol. However, fluorescence microscopy suggested that ixmyelocel-T macrophages do not store large pools of cholesterol esters, suggesting that a majority of this cholesterol remains in the free state ready for efflux.

These data suggest that the macrophages generated by ixmyelocel-T may use protective mechanisms to prevent uncontrolled cholesterol loading, which is toxic to macrophages. These results suggest that ixmyelocel-T macrophages use a protective mechanism against unregulated modified cholesterol uptake by not upregulating scavenger receptor expression in the presence of modified cholesterol, as well as not storing vast amounts as esters. This observation is in alignment with the reduced apoptosis found in ixmyelocel-T macrophages after cholesterol loading and the minimal increase in pro-inflammatory cytokine secretion after lipid loading and inflammatory challenge. The relatively low expression of scavenger receptors after modified cholesterol loading is one potential mechanism by which ixmyelocel-T is protected from cellular apoptosis after high lipid loading.

ACAT-1 promotes accumulation of intracellular CE in macrophages [33]. Studies have demonstrated that ACAT-1 plays an important role in atherosclerosis and is expressed at elevated levels in foam cells located within atherosclerotic lesions [7,33]. In addition, studies have also demonstrated that reduced ACAT-1 expression has been reported to inhibit foam cell formation in macrophages [33]. The results of this study show that a statistically significant decrease in *ACAT-1* expression occurred in ixmyelocel-T macrophages when compared with THP-1 macrophages. Moreover, after loading with Ac-LDL, expression of *ACAT-1* was not increased in ixmyelocel-T macrophages, a feature THP-1 macrophages did not share.

Hydrolysis of stored CE is the first and rate-limiting step in cholesterol efflux from macrophage foam cells, and increased expression of CEH leads to increased mobilization of CE, thereby decreasing lipid accumulation [9]. After loading with modified cholesterol, ixmyelocel-T macrophages exhibited increased *CEH* expression, the expression of which was decreased in THP-1 macrophages. These results may explain why the fluorescence microscopy images indicated that ixmyelocel-T macrophages do not store large pools of cholesterol esters. A decrease in *ACAT-1* and increase in *CEH* would suggest that a majority of the ingested cholesterol remains in the free state, ready for efflux. The combination of both increased *CEH* and decreased *ACAT-1* expression after cholesterol loading may be one mechanism by which ixmyelocel-T macrophages reduce cholesterol accumulation, thus preventing lipotoxicity.

Macrophages maintain cholesterol homeostasis with a balance between influx and efflux pathways [5]. To appreciate the potential impact of ixmyelocel-T macrophages on cholesterol homeostasis, it is essential to consider all aspects of cholesterol homeostasis, including efflux from cells. This investigation demonstrated that ixmyelocel-T macrophages display enhanced cholesterol efflux capacity. Specifically, these data demonstrated that after loading with Ac-LDL, ixmyelocel-T macrophages upregulated expression of both cholesterol transporter genes, *ABCA1* and *ABCG1*. Both of these transporters are critical in the efflux of cholesterol from macrophages. In addition, results from the cholesterol efflux assay revealed that ixmyelocel-T macrophages were not only able to efflux cholesterol to the acceptor ApoAI and HDL^3^, but also to perform this action at an enhanced level. Ixmyelocel-T macrophages were also able to perform this action *in vivo* in the RCT assay.

Taken together, these data highlight the enhanced ability of ixmyelocel-T to promote RCT through cholesterol influx and efflux. In addition, the results highlight one mechanism by which ixmyelocel-T macrophages maintain cholesterol homeostasis.

Atherosclerosis is chronic inflammatory disease that leads to a multitude of diseases, including coronary artery disease, peripheral artery disease, and stroke [34]. Several therapies have been investigated in atherosclerosis, including antiinflammatory drugs, cytokine, and chemokine antagonists. However, no really promising effects on clinical outcomes have been seen on the reduction of atherosclerosis in CAD [34]. Cell therapies have emerged for the treatment of cardiovascular diseases. Several studies have also provided evidence that cell therapies could be a useful treatment in of atherosclerosis, promoting lesion regression and thereby improving vascular health [34-36]. Furthermore, several studies have demonstrated that atherosclerotic lesions contain both M1 and M2 macrophages, providing evidence that M1 macrophages are found in unstable rupture-prone regions of atherosclerotic plaques [37-39]. Therefore, ixmyelocel-T may be potentially beneficial in the treatment of atherosclerotic disease states in which they might promote lesion regression or stabilization through immunomodulation, efferocytosis, and modified cholesterol removal. Treatment with ixmyelocel-T has been investigated in bone regeneration, critical limb ischemia, and ischemic DCM. Further analysis of the subpopulations of cells generated in ixmyelocel-T could highlight new and innovative uses of this multicellular therapy. In this case, further analysis of ixmyelocel-T macrophages suggests that treatment with this cellular therapy might be advantageous in disease states involving atherosclerotic lesions, which would benefit from immunomodulation, efferocytosis, and modified cholesterol removal. Future studies will examine the effects of ixmyelocel-T in preclinical models to determine whether this therapy can affect the atherosclerotic state. For this cell therapy to affect an atheroma, it might need to be present in the lesion itself. Therefore, future studies will examine whether ixmyelocel-T has the ability to infiltrate atherosclerotic lesions or if it must be directly injected into areas where it might have the potential to dampen the immune response, remove necrotic debris, and efflux cholesterol.

## Conclusion

The present data provide evidence that ixmyelocel-T has the potential to become an effective adjuvant in the treatment of diseases due to atherosclerosis. Ixmyelocel-T contains a unique M2-like population of macrophages that are efficient at efferocytosis and maintaining cholesterol homeostasis. The data presented here suggest that ixmyelocel-T macrophages may exert beneficial effects in atherosclerotic disease because they influx cholesterol, remain antiinflammatory in the face of lipid loading and inflammatory challenge, and display enhanced cholesterol efflux capabilities. The multifactorial characteristics of the ixmyelocel-T M2-like macrophages described in this analysis, as well as the companion manuscript presented in *Stem Cell Research and Therapy*, highlight the potential effectiveness of this expanded autologous multicellular therapy in the treatment of atherosclerotic diseases.

## Abbreviations

ACAT: Acyl-CoA:cholesterol acyl-transferase; Ac-LDL: Acetylated low-density lipoprotein; apoA-I: Apolipoprotein A-I; BSA: Bovine serum albumin; cDNA: Complementary DNA; CEH: Cholesterol ester hydrolase; CES: Carboxylesterase; DAPI: 4′-6-diamidino-2-phenylindole; Dil-Ac-LDL: Low-density lipoprotein from human plasma acetylated, DiI complex; FAM: 6-carboxyyfluorescein; FBS: Fetal bovine serum; FC: Free or unesterified cholesterol; GAPDH: Glyceraldehyde 3-phosphate dehydrogenase; HDL: High-density lipoprotein; HEPES: 4-(2-hydroxyethyl) piperazine-1-ethanesulfonic acid; IL: Interleukin; LDL: Low-density lipoprotein; LPS: Lipopolysaccharide; MCP-1: Monocyte chemoattractant protein-1; MEM: Minimal essential medium; PBS: Phosphate-buffered saline; PCR: Polymerase chain reaction; RCT: Reverse cholesterol transport; RNA: Ribonucleic acid; RPMI: Roswell Park Memorial Institute medium; SCARB1: Scavenger receptor class B type I; SCID: Severe combined immunodeficient; SEM: Standard error of the mean; SR: Scavenger receptor; THP-1: Human leukemia monocyte; TNF-α: Tumor necrosis factor-alpha.

## Competing interests

All authors are employees of Aastrom Biosciences, Inc.

## Authors’ contributions

KL conceived and designed research, acquired data, analyzed and interpreted data and results, performed statistical analysis, and drafted the manuscript. NM prepared samples, analyzed data, and reviewed and provided feedback during the development of the manuscript. FZ provided conceptual advice, analyzed data, and participated in discussion of results. RB contributed to the scientific direction, experimental approach, and interpretation of results. All authors read and approved the final manuscript.

## References

[B1] Yao S Zong C Zhang Y Sang H Yang M Jiao P Fang Y Yang N Song G Qin S Activating transcription factor 6 mediates oxidized LDL-induced cholesterol accumulation and apoptosis in macrophages by up-regulating CHOP expression J Atheroscler Thromb 2012 4 94 107 23037953 10.5551/jat.13425

[B2] Hilgendorf I Swirski FK Making a difference: monocyte heterogeneity in cardiovascular disease Curr Atheroscler Rep 2012 4 450 459 10.1007/s11883-012-0274-8 22847772 PMC3436972

[B3] Chinetti-Gbaguidi G Baron M Bouhlel MA Vanhoutte J Copin C Sebti Y Derudas B Mayi T Bories G Tailleux A Haulon S Zawadzki C Jude B Staels B Human atherosclerotic plaque alternative macrophages display low cholesterol handling but high phagocytosis because of distinct activities of the PPARgamma and LXRalpha pathways Circ Res 2011 4 985 995 10.1161/CIRCRESAHA.110.233775 21350215 PMC3319502

[B4] Pou J Rebollo A Roglans N Sanchez RM Vazquez-Carrera M Laguna JC Pedro-Botet J Alegret M Ritonavir increases CD36, ABCA1 and CYP27 expression in THP-1 macrophages Exp Biol Med (Maywood) 2008 4 1572 1582 10.3181/0805-RM-144 18849545

[B5] Ghosh S Macrophage cholesterol homeostasis and metabolic diseases: critical role of cholesteryl ester mobilization Exp Rev Cardiovasc Ther 2011 4 329 340 10.1586/erc.11.16 PMC3098044 21438812

[B6] Moore KJ Tabas I Macrophages in the pathogenesis of atherosclerosis Cell 2011 4 341 355 10.1016/j.cell.2011.04.005 21529710 PMC3111065

[B7] Hongo S Watanabe T Arita S Kanome T Kageyama H Shioda S Miyazaki A Leptin modulates ACAT1 expression and cholesterol efflux from human macrophages Am J Physiol Endocrinol Metab 2009 4 E474 E482 10.1152/ajpendo.90369.2008 19625677

[B8] Sankaranarayanan S de la Llera-Moya M Drazul-Schrader D Asztalos BF Weibel GL Rothblat GH Importance of macrophage cholesterol content on the flux of cholesterol mass J Lipid Res 2010 4 3243 3249 10.1194/jlr.M008441 20713652 PMC2952564

[B9] Bie J Zhao B Ghosh S Atherosclerotic lesion progression is attenuated by reconstitution with bone marrow from macrophage-specific cholesteryl ester hydrolase transgenic mice Am J Physiol Regul Integr Comp Physiol 2011 4 R967 R974 10.1152/ajpregu.00277.2011 21795638 PMC3197345

[B10] Yamamoto S Tanigawa H Li X Komaru Y Billheimer JT Rader DJ Pharmacologic suppression of hepatic ATP-binding cassette transporter 1 activity in mice reduces high-density lipoprotein cholesterol levels but promotes reverse cholesterol transport Circulation 2011 4 1382 1390 10.1161/CIRCULATIONAHA.110.009704 21859969 PMC3323112

[B11] Cuchel M Rader DJ Macrophage reverse cholesterol transport: key to the regression of atherosclerosis? Circulation 2006 4 2548 2555 10.1161/CIRCULATIONAHA.104.475715 16735689

[B12] Suratt BT Fessler MB Greasing the way: the ABCs of HSPC efflux from the marrow Cell Stem Cell 2012 4 143 144 10.1016/j.stem.2012.07.009 22862939 PMC3429340

[B13] Cuchel M Lund-Katz S de la Llera-Moya M Millar JS Chang D Fuki I Rothblat GH Phillips MC Rader DJ Pathways by which reconstituted high-density lipoprotein mobilizes free cholesterol from whole body and from macrophages Arterioscler Thromb Vasc Biol 2010 4 526 532 10.1161/ATVBAHA.109.196105 20018934 PMC2842952

[B14] Bouhlel MA Derudas B Rigamonti E Dievart R Brozek J Haulon S Zawadzki C Jude B Torpier G Marx N Staels B Chinetti-Gbaguidi G PPARgamma activation primes human monocytes into alternative M2 macrophages with anti-inflammatory properties Cell Metab 2007 4 137 143 10.1016/j.cmet.2007.06.010 17681149

[B15] Jennewein C Kuhn AM Schmidt MV Meilladec-Jullig V von Knethen A Gonzalez FJ Brune B Sumoylation of peroxisome proliferator-activated receptor gamma by apoptotic cells prevents lipopolysaccharide-induced NCoR removal from kappaB binding sites mediating transrepression of proinflammatory cytokines J Immunol 2008 4 5646 5652 18832723 10.4049/jimmunol.181.8.5646 PMC2679654

[B16] Mosser DM The many faces of macrophage activation J Leukoc Biol 2003 4 209 212 10.1189/jlb.0602325 12554797

[B17] Deonarine K Panelli MC Stashower ME Jin P Smith K Slade HB Norwood C Wang E Marincola FM Stroncek DF Gene expression profiling of cutaneous wound healing J Transl Med 2007 4 11 10.1186/1479-5876-5-11 17313672 PMC1804259

[B18] Mantovani A Garlanda C Locati M Macrophage diversity and polarization in atherosclerosis: a question of balance Arterioscler Thromb Vasc Biol 2009 4 1419 1423 10.1161/ATVBAHA.108.180497 19696407

[B19] Khallou-Laschet J Varthaman A Fornasa G Compain C Gaston AT Clement M Dussiot M Levillain O Graff-Dubois S Nicoletti A Caligiuri G Macrophage plasticity in experimental atherosclerosis PLoS One 2010 4 e8852 10.1371/journal.pone.0008852 20111605 PMC2810335

[B20] Pello OM Silvestre C De Pizzol M Andres V A glimpse on the phenomenon of macrophage polarization during atherosclerosis Immunobiology 2011 4 1172 1176 10.1016/j.imbio.2011.05.010 21802768

[B21] Hoeksema MA Stoger JL de Winther MP Molecular pathways regulating macrophage polarization: implications for atherosclerosis Curr Atheroscler Rep 2012 4 254 263 10.1007/s11883-012-0240-5 22407286 PMC3348484

[B22] Cardilo-Reis L Gruber S Schreier SM Drechsler M Papac-Milicevic N Weber C Wagner O Stangl H Soehnlein O Binder CJ Interleukin-13 protects from atherosclerosis and modulates plaque composition by skewing the macrophage phenotype EMBO Mol Med 2012 4 1072 1086 10.1002/emmm.201201374 23027612 PMC3491837

[B23] Feig JE Vengrenyuk Y Reiser V Wu C Statnikov A Aliferis CF Garabedian MJ Fisher EA Puig O Regression of atherosclerosis is characterized by broad changes in the plaque macrophage transcriptome PLoS One 2012 4 e39790 10.1371/journal.pone.0039790 22761902 PMC3384622

[B24] Bartel RL Cramer C Ledford K Longcore A Parrish C Stern T Watling S Zeigler F The Aastrom experience Stem Cell Res Ther 2012 4 26 10.1186/scrt117 22776246 PMC3580464

[B25] Thorp E Tabas I Mechanisms and consequences of efferocytosis in advanced atherosclerosis J Leukoc Biol 2009 4 1089 1095 10.1189/jlb.0209115 19414539 PMC2774877

[B26] Auwerx J The human leukemia cell line, THP-1: a multifaceted model for the study of monocyte-macrophage differentiation Experientia 1991 4 22 31 10.1007/BF02041244 1999239

[B27] Napolitano M De Pascale C Wheeler-Jones C Botham KM Bravo E Effects of lycopene on the induction of foam cell formation by modified LDL Am J Physiol Endocrinol Metab 2007 4 E1820 E1827 10.1152/ajpendo.00315.2007 17911344

[B28] Huang Y Ghosh MJ Lopes-Virella MF Transcriptional and post-transcriptional regulation of LDL receptor gene expression in PMA-treated THP-1 cells by LDL-containing immune complexes J Lipid Res 1997 4 110 120 9034205

[B29] Michael DR Salter RC Ramji DP TGF-beta inhibits the uptake of modified low density lipoprotein by human macrophages through a Smad-dependent pathway: a dominant role for Smad-2 Biochim Biophys Acta 1822 4 1608 1616 10.1016/j.bbadis.2012.06.002 PMC3497875 22705205

[B30] Yin D Wang Z Gao Q Sundaresan R Parrish C Yang Q Krebsbach PH Lichtler AC Rowe DW Hock J Liu P Determination of the fate and contribution of ex vivo expanded human bone marrow stem and progenitor cells for bone formation by 2.3ColGFP Mol Ther 2009 4 1967 1978 10.1038/mt.2009.151 19603005 PMC2835035

[B31] Annema W Tietge UJ Regulation of reverse cholesterol transport: a comprehensive appraisal of available animal studies Nutr Metab (Lond) 2012 4 25 10.1186/1743-7075-9-25 22458435 PMC3366910

[B32] Rosenson-Schloss RS Chnari E Brieva TA Dang A Moghe PV Glutathione preconditioning attenuates Ac-LDL-induced macrophage apoptosis via protein kinase C-dependent Ac-LDL trafficking Exp Biol Med (Maywood) 2005 4 40 48 15618124 10.1177/153537020523000105

[B33] Zhao ZZ Wang Z Li GH Wang R Tan JM Cao X Suo R Jiang ZS Hydrogen sulfide inhibits macrophage-derived foam cell formation Exp Biol Med (Maywood) 2011 4 169 176 10.1258/ebm.2010.010308 21321313

[B34] Zenovich AG Taylor DA Atherosclerosis as a disease of failed endogenous repair Front Biosci 2008 4 3621 3636 18508460 10.2741/2954 PMC2666301

[B35] Lima LC Porto ML Campagnaro BP Tonini CL Nogueira BV Pereira TM Vasquez EC Meyrelles SS Mononuclear cell therapy reverts cuff-induced thrombosis in apolipoprotein E-deficient mice Lipids Health Dis 2012 4 96 10.1186/1476-511X-11-96 22849299 PMC3477089

[B36] Porto ML Lima LC Pereira TM Nogueira BV Tonini CL Campagnaro BP Meyrelles SS Vasquez EC Mononuclear cell therapy attenuates atherosclerosis in apoE KO mice Lipids Health Dis 2011 4 155 10.1186/1476-511X-10-155 21896159 PMC3179743

[B37] Oh J Riek AE Weng S Petty M Kim D Colonna M Cella M Bernal-Mizrachi C Endoplasmic reticulum stress controls M2 macrophage differentiation and foam cell formation J Biol Chem 2012 4 11629 11641 10.1074/jbc.M111.338673 22356914 PMC3320912

[B38] Shaikh S Brittenden J Lahiri R Brown PA Thies F Wilson HM Macrophage subtypes in symptomatic carotid artery and femoral artery plaques Eur J Vasc Endovasc Surg 2012 4 491 497 10.1016/j.ejvs.2012.08.005 22975154

[B39] Stoger JL Gijbels MJ van der Velden S Manca M van der Loos CM Biessen EA Daemen MJ Lutgens E de Winther MP Distribution of macrophage polarization markers in human atherosclerosis Atherosclerosis 2012 4 461 468 10.1016/j.atherosclerosis.2012.09.013 23078881

